# Supplementary material for: Plcz1 Deficiency Decreased Fertility in Male Mice Which Is Associated with Sperm Quality Decline and Abnormal Cytoskeleton in Epididymis
Source: Int J Mol Sci. 2022 Dec 24;24(1):314. doi: 10.3390/ijms24010314 (PMC9820195; doi:10.3390/ijms24010314)
Supplement: Supplementary file 1 [file ijms-24-00314-s001.zip › ijms-2039573-supplementary.pdf]

Table S1. Early embryonic development by in vivo fertilization of *Plcz1*$^{-/-}$ mice.

Group	Time/h	1-cell	2-cell	4-cell	8-cell	Morula	Blastocyst	Other
WT	24	100.00%	—	—	—	—	—	—
	48	12.08±14.75%	81.93±17.80%	2.94±6.58%	—	—	—	3.04±3.88%
	72	9.15±9.41%	6.92±6.22%	44.35±27.26%	20.03±19.0%	14.81±16.69%	—	4.75±5.11%
	96	4.72±6.52%	7.02±6.23%	1.83±2.71%	2.60±4.26%	72.03±17.30%	2.72±4.02%	9.07±7.48%
	120	1.66±2.41%	5.19±3.90%	2.98±5.06%	—	21.20±15.10%	53.26±21.53%	15.72±12.76%
Plcz1^{m3}	24	92.44±7.93%	—	—	—	—	—	7.56±7.93%
	48	37.24±20.24%	44.65±12.30%*	1.19±1.68%	—	—	—	16.92±8.99%*
	72	30.19±15.88%	12.50±10.21%	34.89±17.62%	2.13±1.56%	—	—	20.29±10.11%*
	96	24.83±10.80%*	11.01±8.45%	7.64±8.04%	5.65±4.39%	21.76±16.13%**	—	29.10±13.49%*
	120	7.64±5.47%	7.64±5.47%	3.37±3.40%	0.69±0.98%	10.88±8.07%	6.77±8.34%*	63.01±5.54%***
Plcz1^{m5}	24	100.00%	—	—	—	—	—	—
	48	27.54±8.11%	61.09±10.77%	1.10±1.55%	—	—	—	10.27±9.03%
	72	17.38±5.59%	28.17±32.38%	35.73±24.69%	5.27±5.29%	0.73±1.04%	—	12.71±10.79%
	96	4.40±6.22%	26.82±25.86%	8.55±8.29%	—	17.26±15.45%**	—	42.97±13.83%**
	120	1.10±1.55%	15.31±16.51%	9.29±9.29%	—	—	4.40±6.22%*	69.91±21.29%**

Table S2. Early embryonic development by in vitro fertilization of *Plcz1*$^{-/-}$ mice.

Group	Time/h	1-cell	2-cell	4-cell	8-cell	Morula	Blastocyst	Other
WT	24	94.20±8.20%	4.83±6.83%	—	—	—	—	0.97±1.37%
	48	27.26±10.37%	68.99±12.20%	—	—	—	—	3.74±3.45%
	72	20.95±8.83%	2.08±2.95%	70.65±11.53%	—	—	—	6.31±1.63%
	96	9.57±5.02%	1.39±1.96%	2.57±2.67%	—	55.31±5.46%	17.84±12.25%	13.32±4.14%
	120	4.23±3.40%	—	1.39±1.96%	—	23.37±5.52%	53.68±8.33%	17.33±5.48%
Plcz1^{m3}	24	95.16±6.84%	—	—	—	—	—	4.84±6.84%
	48	66.91±6.07%**	24.40±9.47%*	—	—	—	—	8.69±7.25%
	72	60.37±8.76%*	13.54±2.90%*	13.54±2.90%**	—	—	—	12.54±9.04%
	96	41.04±17.43%	3.23±4.56%	5.16±3.68%	—	19.61±12.13%*	1.08±1.52%	29.89±21.28%
	120	16.13±22.81%	5.61±4.10%	5.56±7.86%	—	11.53±7.14%	1.61±2.28%**	59.56±19.36%*
Plcz1^{m5}	24	98.45±2.19%	—	—	—	—	—	1.55±2.19%
	48	61.26±12.20%*	35.09±12.27%	—	—	—	—	3.66±2.86%
	72	43.38±10.94%	30.33±16.59%	12.53±10.45%**	—	—	—	13.77±4.54%
	96	29.50±14.09%	20.22±14.74%	7.10±5.14%	2.11±1.64%	14.43±6.77%**	3.88±5.48%	22.76±6.71%
	120	4.65±6.58%	16.88±10.37%	1.55±2.19%	—	12.43±11.64%	3.88±5.48%**	60.60±6.64%**

Table S3. Sperm motility parameters of *Plcz1*$^{-/-}$ mice.

Group	Forward movement sperm (Grade a+b)		Non-forward movement sperm (Grade c)		Inactive sperm (Grade d)		Sperm activity rate (Grade a+b+c) /%
	Concentration / (10^6 ml^{-1})	Percentage /%	Concentration / (10^6 ml^{-1})	Percentage /%	Concentration / (10^6 ml^{-1})	Percentage /%	
WT	21.64±17.71	48.76±9.19	4.14±2.92	9.95±0.89	16.57±12.65	41.29±9.72	58.71±9.72
Plcz1^{m3}	8.28±5.30**	23.37±9.90**	4.0±3.13	10.14±4.30	25.70±19.87	66.49±10.4**	33.51±10.40**
Plcz1^{m5}	11.53±5.45	46.52±7.39	2.73±1.24	13.19±3.96	9.74±4.17	40.28±9.28	57.22±9.48

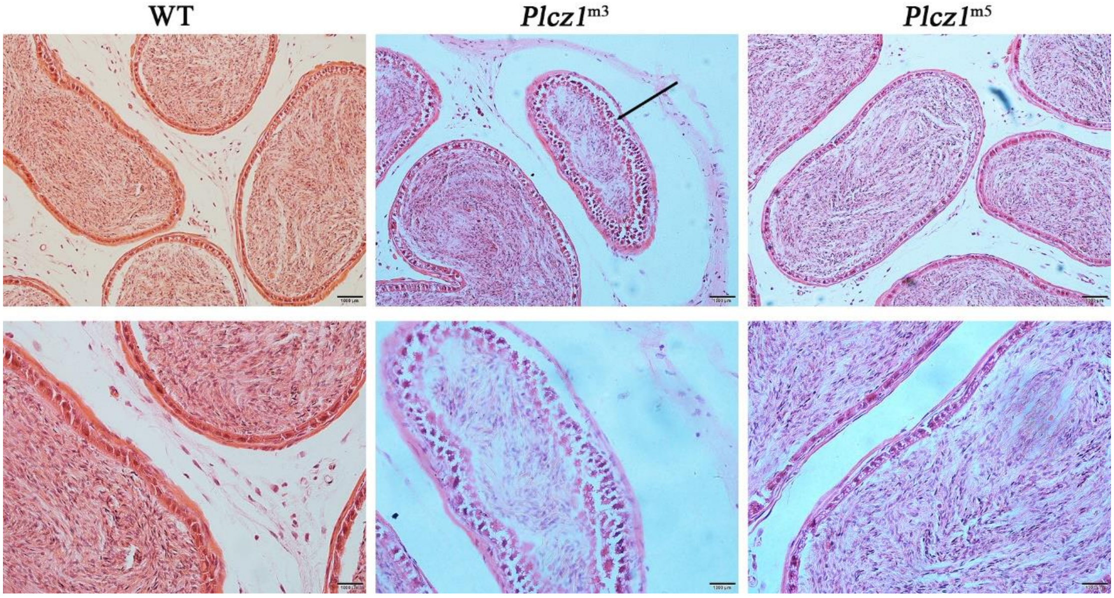

Figure S1. Histopathological changes in the luminal components of the cauda epididymis after stained with HE. Black arrows indicated the shedding epithelial cells in the lumen.

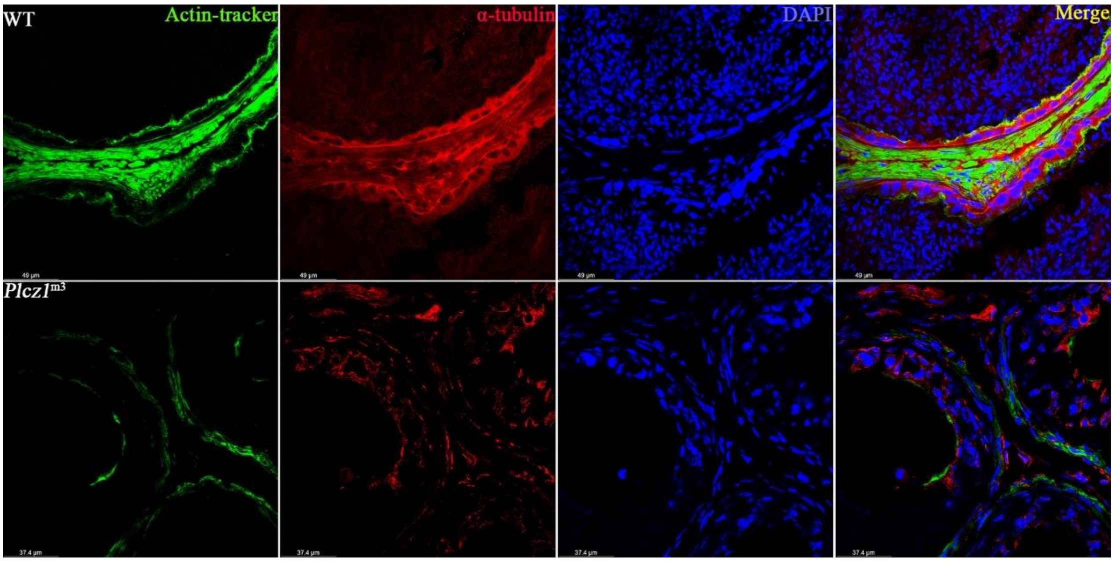

Figure S2. Effects of *Plcz1* knockout on cytoskeleton in cauda epididymis of *Plcz1*^{m3}.
